# Heart Structure-Specific Transcriptomic Atlas Reveals Conserved microRNA-mRNA Interactions

**DOI:** 10.1371/journal.pone.0052442

**Published:** 2013-01-02

**Authors:** Caterina Vacchi-Suzzi, Florian Hahne, Philippe Scheubel, Magali Marcellin, Valerie Dubost, Magdalena Westphal, Catherine Boeglen, Stine Büchmann-Møller, Ming Sin Cheung, André Cordier, Christopher De Benedetto, Mark Deurinck, Moritz Frei, Pierre Moulin, Edward Oakeley, Olivier Grenet, Armelle Grevot, Robert Stull, Diethilde Theil, Jonathan G. Moggs, Estelle Marrer, Philippe Couttet

**Affiliations:** 1 Preclinical Safety, Novartis Institutes of Biomedical Research, Basel, Switzerland; 2 Biomarker Development, Novartis Institute for Biomedical Research, Basel, Switzerland; 3 Preclinical Safety, Novartis Institute of Biomedical Research, East Hanover, New Jersey, United States of America; Tokai University, Japan

## Abstract

MicroRNAs are short non-coding RNAs that regulate gene expression at the post-transcriptional level and play key roles in heart development and cardiovascular diseases. Here, we have characterized the expression and distribution of microRNAs across eight cardiac structures (left and right ventricles, apex, papillary muscle, septum, left and right atrium and valves) in rat, Beagle dog and cynomolgus monkey using microRNA sequencing. Conserved microRNA signatures enriched in specific heart structures across these species were identified for cardiac valve (miR-let-7c, miR-125b, miR-127, miR-199a-3p, miR-204, miR-320, miR-99b, miR-328 and miR-744) and myocardium (miR-1, miR-133b, miR-133a, miR-208b, miR-30e, miR-499-5p, miR-30e*). The relative abundance of myocardium-enriched (miR-1) and valve-enriched (miR-125b-5p and miR-204) microRNAs was confirmed using in situ hybridization. MicroRNA-mRNA interactions potentially relevant for cardiac functions were explored using anti-correlation expression analysis and microRNA target prediction algorithms. Interactions between miR-1/Timp3, miR-125b/Rbm24, miR-204/Tgfbr2 and miR-208b/Csnk2a2 were identified and experimentally investigated in human pulmonary smooth muscle cells and luciferase reporter assays. In conclusion, we have generated a high-resolution heart structure-specific mRNA/microRNA expression atlas for three mammalian species that provides a novel resource for investigating novel microRNA regulatory circuits involved in cardiac molecular physiopathology.

## Introduction

MicroRNAs are short non-coding RNAs (∼22 nucleotides) encoded by the genome and conserved throughout the evolution of higher eukaryotes. MicroRNAs regulate gene expression at the post-transcriptional level by directing the RISC complex to target mRNAs resulting in translational inhibition and mRNA decay [1]. The expression levels of many genes can be influenced by microRNAs [2]. However, the lack of perfect complementary between microRNAs and their target mRNAs jeopardizes the accurate prediction of mRNA targets, and *in silico* prediction tools need further optimization [3].

During development, microRNA cellular pools are highly dynamic, tuned by temporal and spatial cues [4], [5], [6], [7]. Accumulating evidence implicates microRNAs in numerous physiological and pathological processes, as well as responses to xenobiotics, including drug-induced cardiotoxicity [8], [9], [10], [11], [12], [13], [14], [15], [16]. In particular, several microRNAs that are preferentially expressed in different types of muscles (e.g. miR-1, miR-133, and the myomiRs miR-208, miR-208b and miR-499) play a pivotal role in maintenance of cardiac function [17], [18], and the ablation of microRNAs-RISC machinery can have dramatic effects on cardiac development [19], [20], [21].

Drug-induced cardiac toxicity, which is often irreversible, ranks among the most frequent reasons for compound attrition due to safety liabilities during pharmaceutical development. Gene expression profiling provides a powerful approach for investigating early molecular mechanisms that lead to overt drug-induced cardiac histopathology [22], [23]. In principle, given the central role played by microRNAs in post-transcriptional gene regulation, a change in the level of a specific microRNA may be prodromal to changes in expression of target mRNA transcripts, suggesting that integrated mRNA/microRNA expression profiling may provide novel insights into early drug-induced molecular responses.

Although the conservation of microRNA sequences across species has been thoroughly studied, systematic data on the degree of similarity of microRNA distribution within complex organs and/or tissues across mammalian species are still lacking. A tissue structure-specific microRNA expression atlas would provide a valuable resource for investigating microRNA/mRNA interactions, their molecular functions and the potential tissue structure-specific origin of candidate circulating microRNA biomarkers.

**Figure 1:**
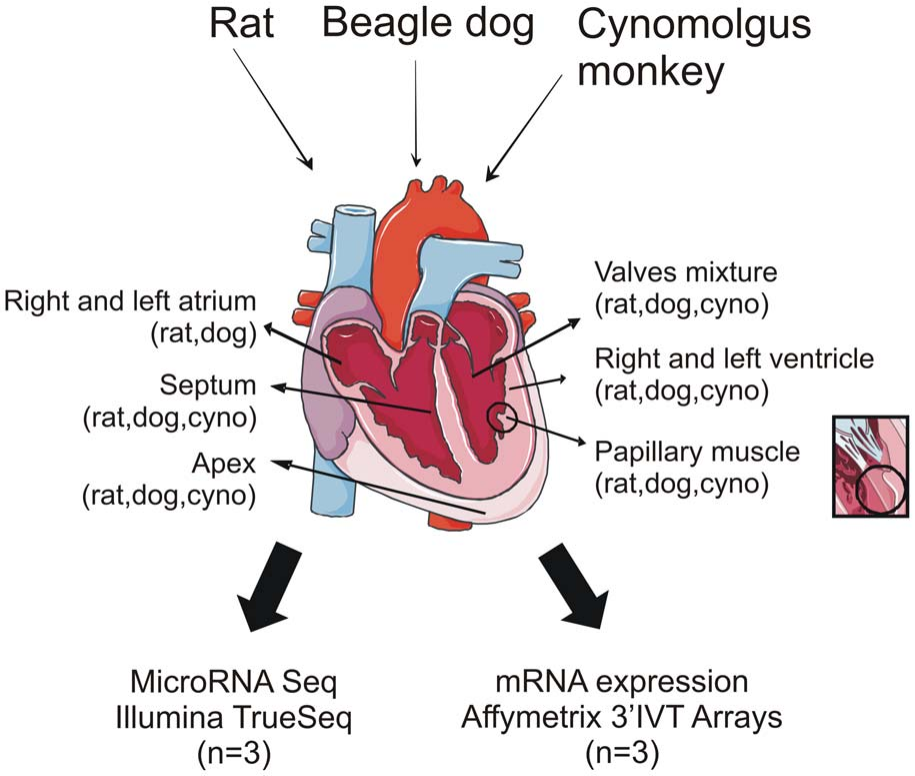
Cross-species microRNA/mRNA cardiac atlas. Data sets obtained for rat, dog and cynomolgus monkey are indicated. Inset: magnified view of a papillary muscle.

Here, we have generated comprehensive microRNA and mRNA expression profiles from 8 cardiac structures, including apex, left and right ventricular walls, papillary muscle, septum, left and right atrial walls and cardiac valves (Figure 1), from three mammalian species that are commonly used in biomedical research (*Rattus norvegicus*, *Canis familiaris*, *and Macaca fascicularis*). Conserved microRNA signatures enriched in specific heart structures across these three species were identified for cardiac valve and myocardium. We have also identified novel microRNA-mediated post-transcriptional mRNA regulatory interactions with potentially important roles in cardiac/muscle physiopathology including miR-1/Timp3, miR-125b/Rbm24, miR-204/Tgfbr2 and miR-208b/Csnk2a2.

The data presented here provide a comprehensive structure-specific transcriptomic atlas of the cardiac organ for three mammalian species that will facilitate the investigation of microRNA/mRNA regulatory interactions in cardiac physiopathology mechanisms and may also enhance the identification of cardiac tissue injury biomarkers.

## Results

### Distribution of microRNAs and mRNAs in Specific Cardiac Structures

**Figure 2:**
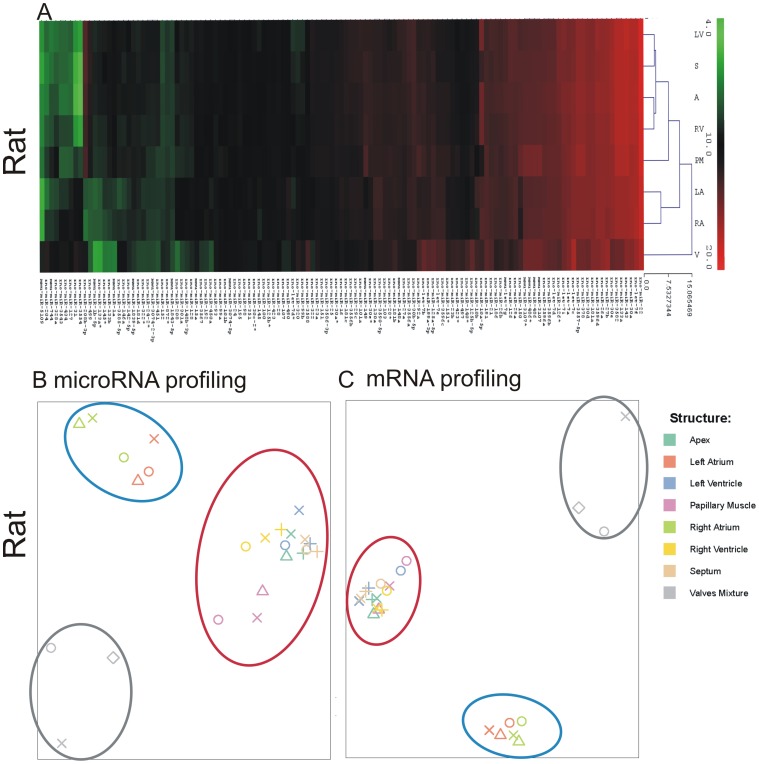
Distribution of microRNAs and mRNAs in rat cardiac structures. (A) Cardiac samples are grouped according to the structure using microRNA signatures (top 10%) in a hierarchical Euclidean-linkage clustering based on miRBase17 mapping (B) Principal Component Analysis of all microRNA profiles based on miRBase17 mapping. (C) Principal Component Analysis of all mRNA profiles. Red circles: myocardial tissue (apex, left and right ventricle, septum, papillary muscle). Blue circles: left and right atrium. Grey circles: cardiac valves. A, apex. LA, left atrium. LV, left ventricle. PM, papillary muscle. RA, right atrium. RV, right ventricle. S, septum. V, valves. Individual animals are indicated by symbols.

Between 260 and 340 unique mature microRNAs were found expressed (normalized sequencing counts >10) in each cardiac structure from the three different species. Hierarchical Euclidean linkage clustering of cardiac microRNA profiles revealed 3 distinct clusters comprising the two ventricles, apex, septum and papillary muscles (myocardium), the two atria (atria) and the cardiac valves (Figure 2A). Similarly, principal component analysis of the rat microRNA and mRNA data sets showed that individual samples from these three main structures clearly grouped together (Figure 2B and C). Valves were clearly separated from atrial and ventricular samples by both microRNA and mRNA profiles, while ventricles, septum, papillary muscles and apex were virtually indistinguishable from each other and therefore pooled together for further analysis under the name of “myocardium”. The similarity of “myocardium” transcript profiles is consistent with the predominance of cardiomyocytes in these structures. Left and right atria profiles were also combined in further analyses based on the similarity of their profiles.

The heart structure-specific clusters obtained in rat were reproducible and consistent for the dog and cynomolgus monkey, suggesting that the structure-specific signatures were robust and unaffected by inter-individual animal differences (Figure S1).

### Conserved Structure-enriched microRNAs and mRNAs Across Species

Since the quality of microRNA annotation contained in miRBase varies greatly between the different species, the microRNA reference libraries were augmented by microRNA definitions from closely related species to broaden the potential search space. In particular, the rat microRNA library was augmented by mouse microRNA definitions, and both the dog and the cynomolgus libraries by human microRNA definitions. To be able to compare microRNA expression values across different species, microRNA paralogs were identified based on the miRBase identifiers and the actual microRNA sequences. Structure-enriched mRNAs were also identified by applying a similar analytical process (see Data Analysis section in Materials and Methods for details).

**Figure 3:**
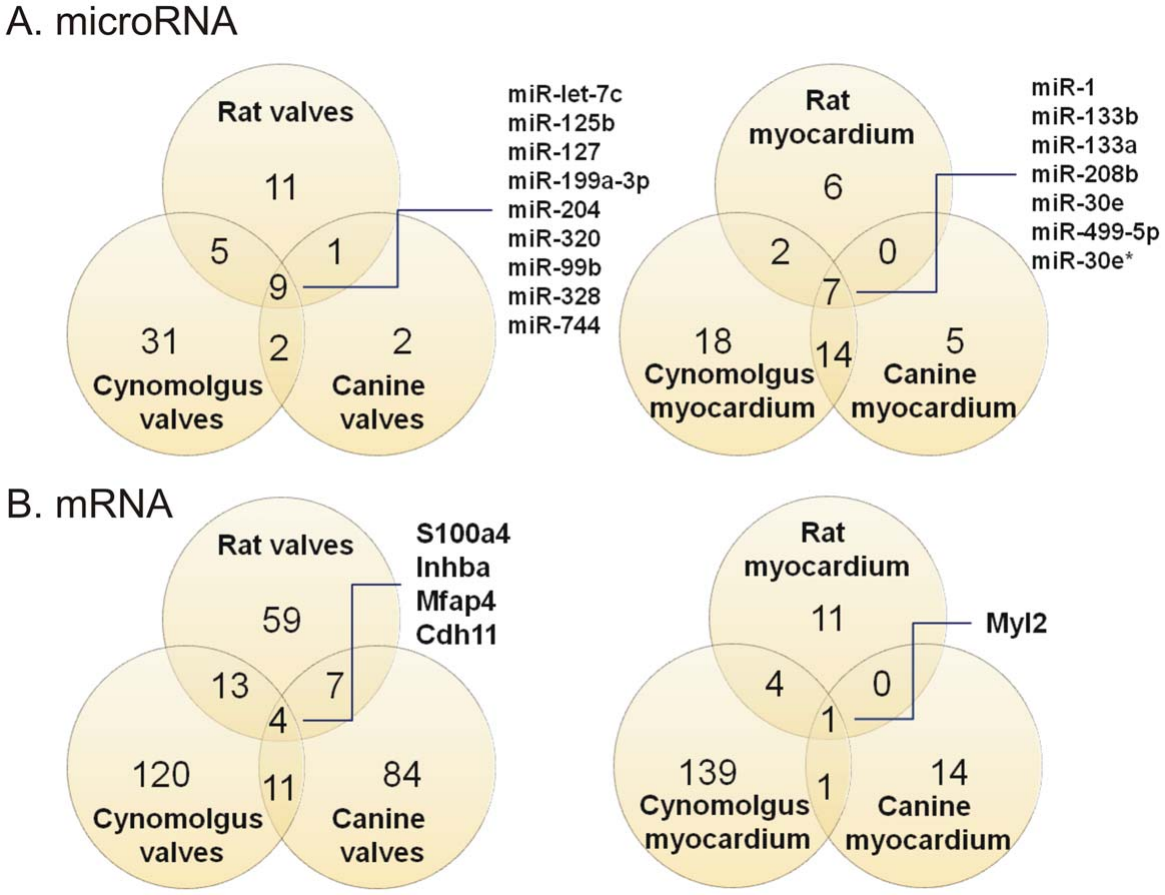
Number of microRNAs (A) and mRNAs (B) preferentially expressed in cardiac valves or myocardium across species. Myocardium includes apex, ventricles, septum and papillary muscles. Fold change thresholds were > = 2 for microRNAs in (A), and > = 5 from mRNAs in (B).

MicroRNAs and mRNAs that were preferentially enriched in only one major cardiac structure (i.e. valves, myocardium or atria) were identified for each of the three species studied (Table S2 for rat, S3 for dog and S4 for cynomolgus monkey). We propose that these transcripts are likely to play important roles in cardiac structure-specific gene expression homeostasis because of their high basal expression level. An assessment of the degree of conservation for structure-specific distribution of microRNAs in Wistar rat, Beagle dog and cynomolgus monkey (see Materials and Methods for relative enrichment analysis), revealed high enrichment of nine microRNAs cardiac valves (miR-let7c, mIR-125b, miR-127, mir-199a-3p, miR204, miR-320, miR-99b, miR-328 and miR-744) (Figure 3A) and seven microRNAs in the myocardium (miR-1, mir-133a, miR-133b, miR-208b, miR-30e, miR-499-5p, miR-30e*) (Figure 3A). At the mRNA level, the expression of four transcripts (S100a4, Inhba, Mfap4 and Cdh11) was enriched in cardiac valves whilst Myl2 mRNA expression was enriched in myocardium (Figure 3B). The presence of predicted microRNA binding sites within the mRNA sequence of these five cardiac structure-enriched genes was explored further. Notably, the cardiac valve-enriched Cdh11 mRNA contains a miR-27a binding site in rat, dog, cynomolgus monkey and human. In rat and monkey, miR-27a is enriched in the myocardium and its expression profile is anti-correlated to Cdh11 with a coefficient of −0.67 and −0.76 (Tables S5 and S7). Interestingly, Cdh11 has been shown to play a role in the cardiac valve development [24].

**Figure 4:**
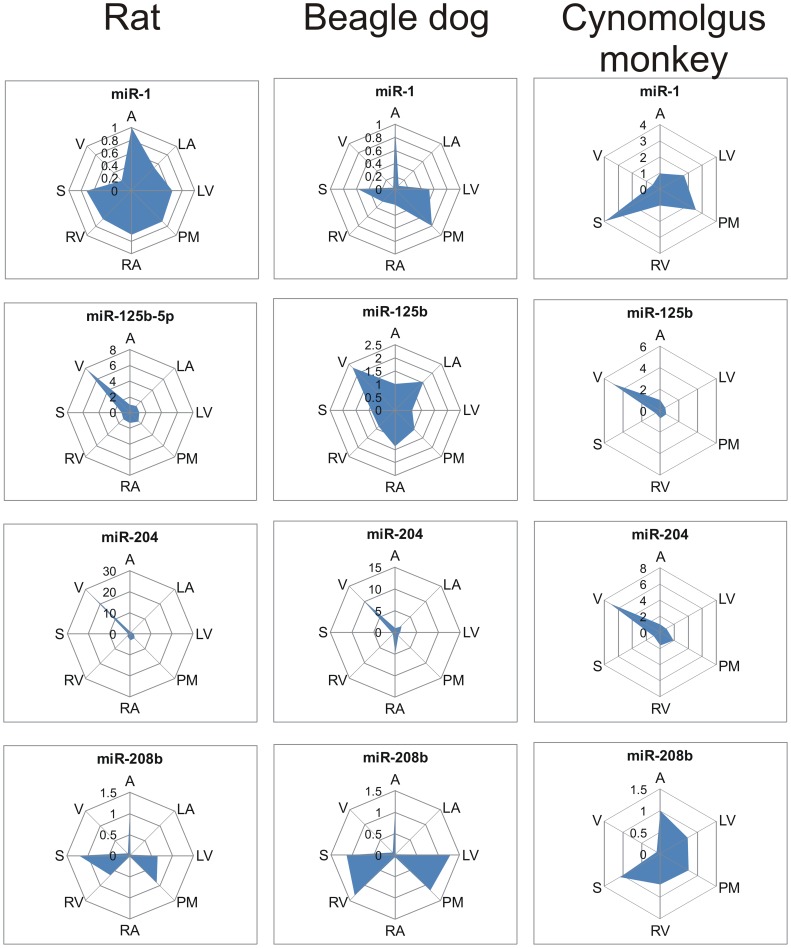
Distribution of miR-1, miR-125b-5p, miR-204 and miR-208b in cardiac structures across species. Axes represent fold change vs. apex. A, apex; LA, left atrium; LV, left ventricle; PM, papillary muscle; RA, right atrium; RV, right ventricle; S, septum; V, valve.

A subset of microRNAs (miR-1, miR-125b-5p, miR-204 and miR-208b) was selected for further cross-species analysis and their expression level relative to heart apex is shown in Figure 4. MiR-1 was highly expressed in all rat, dog and cynomolgus monkey heart structures except valves. MiR-208b expression was high in myocardium structures but very low in the atria of rat and dog and in valves of all the three species. MiR-125b-5p and miR-204 were highly enriched in the valves of all 3 species compared to all other structures although it is noteworthy that significant miR-125b-5p expression was observed in non-valve structures in dog. Similar structure-specific expression patterns of these microRNAs were observed in heart tissue from a healthy human donor (Figure S6).

### Confirmation of microRNAs Distribution by *in situ* Hybridization

**Figure 5:**
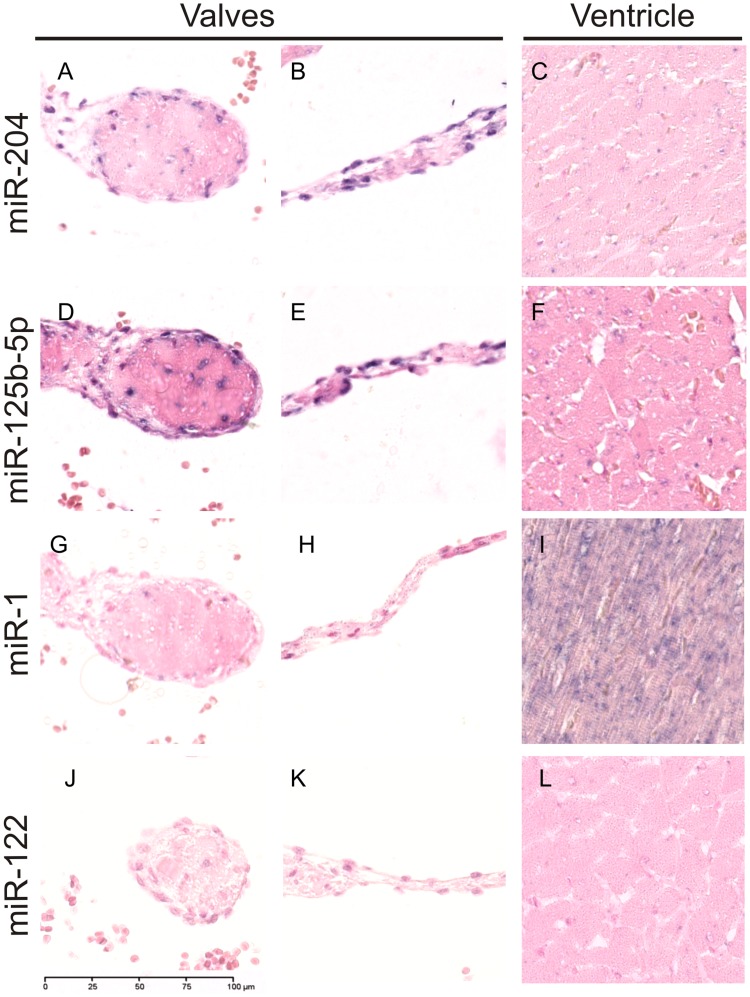
Localization of miR-204, miR-125b-5p, miR-1 and miR-122 in rat heart by *in situ* hybridization. miR-204 in valves (A–B) and ventricle (C). miR-125b-5p in valves (D–E) and ventricle (F). miR-1 in valves (G–H) and ventricle (I). miR-122 in valves (J–K) and ventricle (L). Bar = 100 µm.

MiR-1, miR-204 and miR-125b were detected in rat cardiac tissue by *in situ* hybridization (ISH), and the staining patterns observed were consistent with the relative expression observed by microRNA sequencing and qPCR. The ISH signal for miR-1 was more intense in the myocardium than in the valves (Figure 5G, H and I), and staining for miR-204 and 125b-5p was more intense in the valves than in the rest of the heart (Figure 5A to F), ISH of the liver-enriched miR-122 was performed and used as a negative control (Figure 5J, K and L). Staining for miR-1 was intense and uniform in the cardiomyocytes of the ventricle, while no signal could be detected in the cardiac valves (Figure 5G, H and I and Table S2). No signal for miR-204 was detected in the myocardium (Figure 5C), while valvular endothelial cells were clearly stained (Figure 5A and B) under the same conditions. The specificity of miR-204 for endothelial cells warrants further investigation via ISH of endothelial cell-rich tissues.

Similarly to miR-204, miR-125b-5p showed a strong signal in the cardiac valves and could not be detected in the ventricular cardiomyocytes (Figure 5D, E and F). While signals for miR-1 and miR-125b-5p were strong, miR-204 was at the limit of detection, consistent with its relatively low abundance as determined by microRNA sequencing (Table S8). Attempts to stain miR-208b via ISH were unsuccessful.

In conclusion, the results of the *in situ* hybridization correlated very well with the relative distribution of microRNAs observed via microRNA sequencing (Table S8) and support the specificity of the broader heart structure-specific microRNA profiles described in this study (Tables S2,S3,S4).

### Integration of Cardiac Gene Expression and microRNA Profiles

MicroRNAs targets were obtained from Targetscan and a human database was used for cynomolgus monkey. Spearman correlation scores for each microRNA and their predicted mRNAs targets were calculated across all samples in order to identify mRNA distribution profiles mirroring targeting microRNA counterparts (anti-correlated). Tables S5, S6 and S7 list Spearman correlation scores for the entire microRNA “targetomes” based on our matching datasets in rat, dog and cynomolgus monkey.

**Figure 6:**
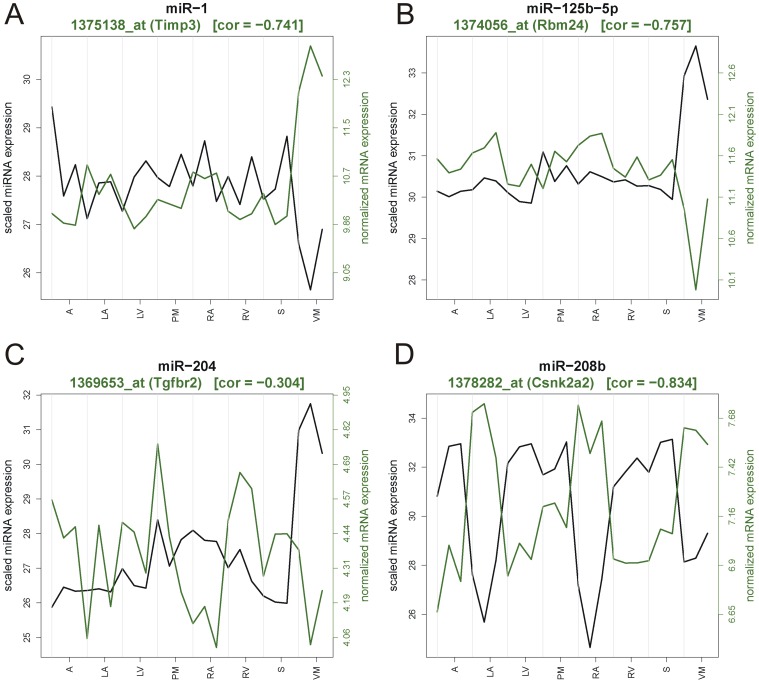
Negative correlation of cardiac tissue microRNA and target mRNA profiles. Timp3 and miR-1 (A), Rbm24 and miR-125b-5p (B), Tgfbr2 and miR-204 (C), Csnk2a2 and miR-208b (D). Green curve represents log2 normalized intensity for the indicated probe set. Black curve represents log2 scaled and normalized microRNA read counts. Three replicates are plotted for each structure. A, apex; LA, left atrium; LV, left ventricle; PM, papillary muscle; RA, right atrium; RV, right ventricle; S, septum; VM, valve.

We focused our attention on mRNAs that had anti-correlated values <−0.7 with respect to microRNAs that had high heart structure specificity, and that were already reported to be implicated in cardiac physiology and/or muscular differentiation. We selected 4 genes (Timp3, Rbm24, Tgfbr2 and Csnk2a2), respectively targeted by miR-1, miR-125b, miR-204 and miR-208b, for further analysis. Their anti-correlated distributions are shown in Figure 6 for the rat. Expression profiles of miR-1/Timp3 and miR-125b-5p/Rbm24 were also anti-correlated with microRNA expression in the dog (Figure S2) and cynomolgus monkey (Figure S3). In contrast, expression profiles of miR-204/Tgfbr2 in canine and cynomolgus monkey, as well as miR-208b/Csnk2a2, in cynomolgus monkey were positively correlated. Interestingly Timp3, Rbm24, Tgfbr2 and Csnk2a2 have previously been implicated in cardiac physiology and/or muscular differentiation [25], [26], [27], [28], [29], [30], [31]. Furthermore, the sequence of predicted microRNA binding sites in the 3′UTRs of Timp3, Rbm24, Tgfbr2 and Csnk2a2 were conserved across species, strengthening the hypothesis that these miR-mRNA interactions could be relevant for cardiac biology (Figure S5).

Human Timp3 was down-regulated by miR-1 over expression in HeLa cells by Lim and colleagues [2], but the authors did not further investigate whether the effect was directly mediated by miR-1 at the post-transcriptional level. Interestingly, Tgfbr2 was shown to be directly targeted by miR-204 in human epithelial cells [32], providing us with a suitable positive control for further confirmatory experiments. Human pulmonary smooth muscle primary cells (HPASM cells) were used to validate a subset of our predicted cardiac tissue microRNA/mRNA interactions. HPASM cells were selected since they express most of the miR-204 pathway components [33], together with the mRNA targets Timp3, Rbm24 and Csnk2a2 mRNA, and provide a model for assessing the translatability of predicted miR/mRNA interactions derived from rat, dog and monkey heart tissues.

**Figure 7:**
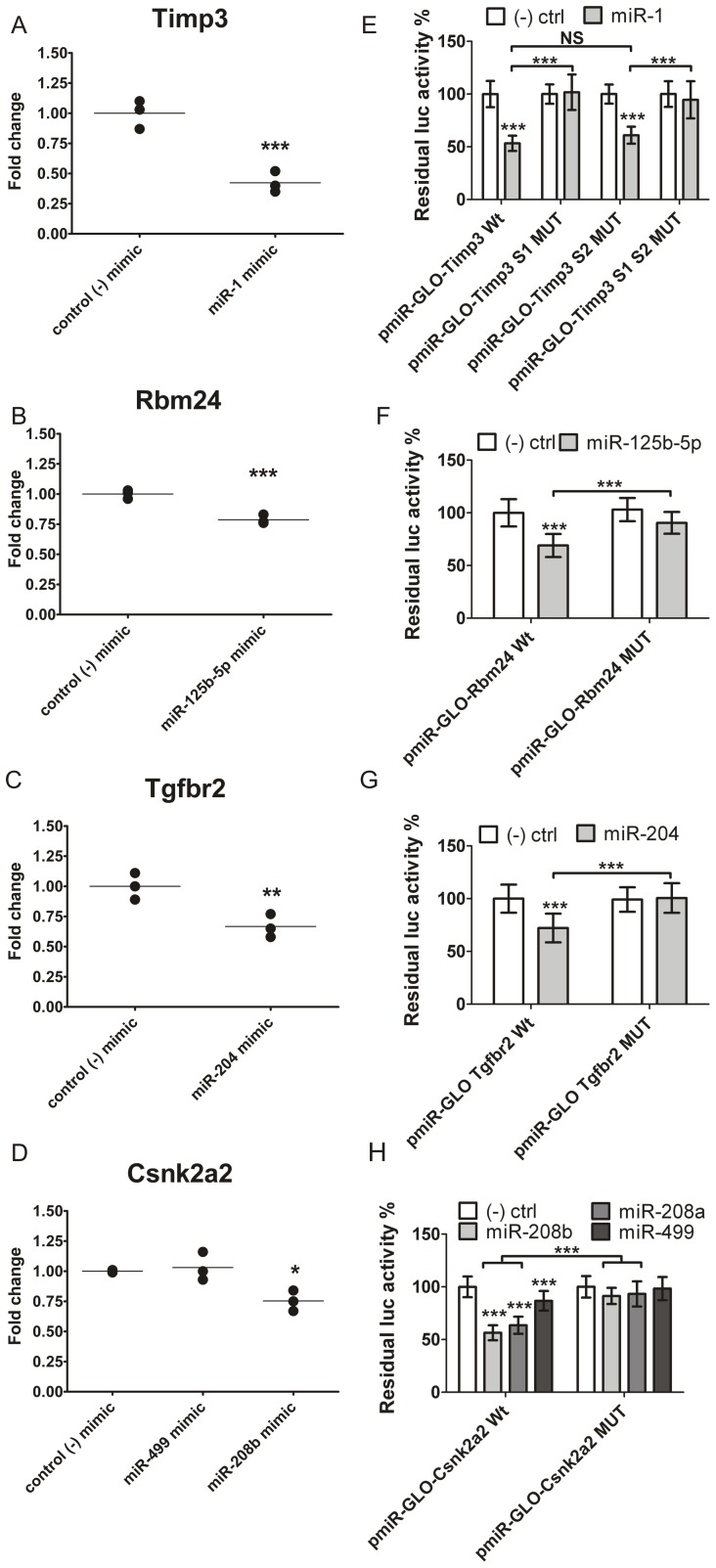
Anti-correlated microRNA targets are directly inhibited by microRNA over expression. (A–D) Real-Time RT-PCR of Timp3, Rbm24, Tgfbr2 and Csnk2a2 in HPASM cells transfected with mimics for miR-1, miR-125b-5p, miR-204, miR-499 and miR-208b or with a mimic microRNA negative control. Data were normalized to 18S RNA. (E-H) Luciferase activity of wild-type (WT) or mutant (MUT) Timp3, Rbm24, Tgfbr2 and Csnk2a2 3′-UTR reporter genes cotransfected with their paired microRNA mimics. Data were averaged for n = 4 in 3 independent experiments and error bars represented standard deviation of the mean. P values: * = P<0.05, ** = P<0.01, *** = P<0.005.

Transfection of miR-1 in HPASM cells resulted in a decrease of about 50% of both the endogenous Timp3 mRNAs level (Figure 7A) and of the corresponding rodent Timp3 3′UTR reporter assay in HEK 293 (Figure 7E). Interestingly, we demonstrated that only one of two predicted miR-1 binding sites within Timp3 was active. The seed mutation of Timp3 miR binding site 1 (pmiR-GLO-Timp3-S1-MUT), but not site 2 (pmiR-GLO-Timp3-S2-MUT), could rescue expression of the reporter gene luciferase, suggesting that site 2 is not involved in the regulation of this gene by miR-1. Limana *et al.* [26] previously reported that the site 2, but not site 1, was targeted by miR-206, which has identical 5′ seed to miR-1, whilst site 1 did not respond to miR-206 over expression. It is noteworthy that miR-206 was not detected in any of the cardiac structures of any of the 3 species investigated in this study, consistent with the observations of Rao *et al.* [34].

The function of Rbm24 and Rbm38 in skeletal muscle is to stabilize the mRNAs of myogenin and p21, respectively [25], [28]. The levels of Rbm24 and Rbm38 mRNAs increased during myoblastic differentiation concomitantly with a decrease in miR-125b-5p expression [35]. Interestingly, Rbm38 was also anti-correlated to miR-125b in our cardiac tissue samples, and has a predicted miR-125b targeting site in its 3′UTR. However only Rbm24 expression was directly inhibited by miR-125b co-transfection in the luciferase assay (Figure 7F), while luc-Rbm38 signal was unaffected (data not shown). Transfection of miR-125b-5p in HPASM cells significantly reduced Rbm24 levels by 25% (Figure 7B), consistent with the effect observed in HEK293 cell 3′UTR reporter assays (Figure 7F). Mutation of the predicted miR-125b-5p binding site within Rbm24 mRNA resulted in rescue of luciferase gene activity.

Transfection of mir-204 significantly decreased the level of Tgfbr2 mRNA by 20% in HPASM cells (Figure 7C) and in the corresponding HEK293 cell-based Tgfbr2 3′ UTR luciferase reporter assay (Figure 7G).

MiR-208b over-expression led to a 25% decrease in Csnk2a2 mRNA levels in HPASM cells (Figure 7D), and a 50% inhibition of luciferase activity in HEK cell reporter assays (Figure 7H). Although the 3 myomiRs miR-208a/miR-208b/miR-499 contain almost identical seed sequences, miR-499 was considerably less potent at inhibiting luc-Csnk2a2 expression than miR-208a/miR-208b, suggesting a functional role for 3′ compensatory interactions between the myomiRs and Csnk2a2 (Figure 7D and H).

In summary, we have demonstrated that four genes (Timp3, Rbm24, Tgfbr2 and Csnk2a2) important for cardiac/muscular physiology are post-transcriptionally regulated by miR-1, miR-125b-5p, miR-204 and miR-208b and exhibit conserved cardiac tissue miR-mRNA interactions across species.

## Discussion

The lethality of a tissue-specific deletion of Dicer in the murine myocardial lineage demonstrates the pivotal role played by microRNAs in cardiovascular development [21]. In particular, miR-1 and miR-133, which are abundant microRNAs in the heart, are implicated in cardiovascular development and myocardial lineage differentiation, as they tightly control expression of muscle genes and repress ”unwanted” gene transcription through a network of target transcription factors [36], [37], [38], [39]. It is noteworthy that miR-1, miR-133, miR-30, miR-208a, miR-208b, mir-499, miR-23a, miR-9 and miR-199a have previously been shown to be functionally involved in cardiovascular diseases such as heart failure and hypertrophy [40], [41], [42], [43], [44], and have been proposed as therapeutic- or disease-related drug targets [45], [46]. However one of the challenges associated with studying the function of specific genes/microRNAs in the cardiac organ is the heterogeneity of cell types and morphology. In order to explore the significance of specific microRNAs in the physiology of distinct cardiac structures we have generated a comprehensive heart structure-specific transcriptomic resource for three mammalian species that will facilitate the investigation of microRNA/mRNA regulatory interactions in cardiac physiopathology mechanisms and may also enhance the identification of cardiac tissue injury biomarkers.

Conserved microRNA signatures were identified in valves (miR-let-7c, miR-125b, miR-127, miR-199a-3p, miR-204, miR-320, miR-99b, miR-328 and miR-744) and in ventricular-specific regions of the myocardium (miR-1, miR-133b, miR-133a, miR-208b, miR-30e, miR-499-5p, miR-30e*) of Wistar rat, Beagle dog and cynomolgus monkey. Conversely, microRNAs that exhibit a dissimilar heart-structure specific distribution across species are likely to be of high interest for translational science applications including the drug cardiac safety assessment.

Here we focused on the characterization of four microRNAs, including myocardial specific miR-1 and miR-208b and valve enriched mir-204 and miR-125b-5p, based on their distinct heart-structure-specific distribution patterns and known roles in cardiac physiology, disease and pathological remodeling. Interestingly, the tissue distribution of each of these four microRNAs was conserved in rat, dog and cynomolgus monkey and human (data from one individual donor are shown in Figure S6). Numerous putative predicted target genes were found to have an anti-correlated distribution with microRNAs across cardiac tissues, as shown in Table S5, S6 and S7, supporting the biological relevance of predicted microRNA-mRNA interactions. However, only a relatively small fraction of each individual microRNA “targetome” had an anti-correlated pattern consistent across samples, emphasing the importance of experimental validation of predicted miRNA-mRNA interactions.

To further investigate the potential significance of predicted mRNA/microRNA interactions derived from rat, dog and monkey cardiac tissues, we evaluated a subset of miR/mRNA interactions in a human cellular model (HPASM) and in HEK 293 luciferase reporter assays. We demonstrate that miR-1 can directly regulate Timp3 expression at the post-transcriptional level. Timp3 is a regulator of adult myogenesis implicated in the fibrotic pathological remodeling of infarcted heart, and in atrial fibrillation [26], [27], [47], [48]. Interestingly, Timp3 has two predicted binding sites for the miR-1/206 family and despite miR-1 and miR-206 (an already known regulator of Timp3) having identical seed sequences, miR-206 specifically targets the second site [26], while miR-1 specifically targets the first site (Figure 6). These observations suggest that duplication of microRNA targeting sites may be a strategy for redundant regulation in cardiac cell types having different microRNA repertoires.

RNA binding motif proteins 24 and 38 (Rbm24 and Rbm38) play a role in muscle differentiation [25], [28] and carry a miR-125b-5p targeting site. Interestingly, the function of miR-125b in controlling proliferation/differentiation switches has been characterized in a number of cell types including immune system and muscular cells [35], [49], [50], [51], [52]. We found that miR-125b-5p over-expression could lower Rbm24, but not Rbm38, levels in HPASM cells, and that the down-regulation was directly mediated by the microRNAs as verified *via* luciferase reporter assay.

TGF-β receptor 2 (Tgfbr2) has been functionally associated with cardiovascular diseases [30], [32], [48], [53] and miR-204 was observed to directly inhibit Tgfbr2 mRNA in a direct manner both in HPASM cells and in a luciferase assay, thus extending the potential roles for miR-204 in cardiac pathogenesis.

Casein kinase 2a2 (Csnk2a2) and miR-208ab have both previously been implicated in cardiovascular pathologies [29], [40], [54] and our data provide further support for cardiac tissue miR208-Csnk2a2 interactions based on their anti-correlated (<−0,8) expression profiles.

The generation of a high-resolution heart structure-specific mRNA/microRNA expression atlas for three mammalian species also provides a novel resource for supporting the identification of diagnostic and drug safety biomarkers. Circulating microRNAs can easily be quantified in body fluids using assays that are fully translatable across species based on the sequence conservation [55] and numerous publications support the use of tissue-specific microRNAs in the peripheral circulation as biomarkers of tissue injury [55], [56], [57], [58]. Heart tissue microRNAs involved in drug-induced cardiac injury may also provide earlier safety endpoints since their altered expression can be prodromal to the corresponding target gene/protein variations [22]. Furthermore, ventricular microRNAs (miR-1, miR-133, miR-208b and miR-499) have been found to be increased in the plasma of patients with myocardial infarction, and might represent a useful alternative to the classical cardiac troponin (cTnI) biomarker [57], [58], [59], [60], [61]. We describe here for the first time a panel of conserved valve-enriched microRNAs that may help to monitor or predict valvular disease and drug-induced valvular injury, for which no translational circulating biomarkers currently exist [59], [62].

The identification of microRNAs that are highly specific for a cell type or morphological structure is challenging, as most of them are ubiquitously expressed, but this may be overcome by measuring a signature of several tissue-enriched microRNAs. This strategy can help to discriminate between closely related tissues (such as skeletal *vs.* cardiac muscle). Combinations of tissue-enriched circulating microRNA biomarkers may also help to monitor tissue specific toxicities.

In conclusion, the data presented here provide a valuable resource to investigate potential roles of specific microRNAs as conserved regulators of cardiac cell homeostasis and heart tissue pathological remodeling. Our data sets can be further analyzed to answer a variety of open questions, such as cell type specific gene/microRNA distribution, involvement of microRNAs in genome plasticity, disease and cellular homeostasis and co-regulation between intronic microRNAs and their hosting transcripts. One important future direction will be to obtain additional clinical samples to confirm the translatability of our preclinical heart structure-specific microRNA profiles and to extend this molecular atlas resource to human cardiac structures. A similar transcriptomic atlas strategy can also be designed for other heterogeneous organs in order to characterize potential structure-specific specific gene regulatory mechanisms and biomarkers.

## Materials and Methods

### Animals and Heart Tissue Dissection

Hearts were obtained from naïve adult (3 to 6 months old) male Wistar Han rats. Following brief infusion with saline, a first cut was made parallel to the auricles, adjacent to the level of the atria; both portions were again rinsed with saline, in order to remove most of the blood. Cardiac valves (mitral and tricuspid) were dissected first, followed by atria (left and right), septum, ventricles (left and right) and apex. Rat valves were pooled together in order to obtain sufficient material for transcriptomic and microRNA sequencing experiments. All tissues were snap frozen in liquid nitrogen and stored at −80°C until RNA isolation. Similar procedures were carried out for male adult (25 to 37 months old) Beagle dogs (*Canis familiaris*) and female adult (2–4 years old) cynomolgus monkeys (*Macaca fascicularis*), except that the different valves were analyzed separately. Rats were sacrificed by CO_2_ inhalation; dogs received a lethal injection of Pentothal (Abbott), while cynomolgus monkeys were first anesthetized with Ketamine/Xylazine, and then received an injection of Euthasol (Virbac Animal Health). All animals were finally sacrificed by exsanguination. All animal investigations were conducted in compliance with the Swiss Animal Welfare Law and Animal Licenses provided by ‘Kantonales Veterinäramt Baselland’ (Baselland, CH) [63] and with the Animal Welfare Act and the Office of Laboratory Animal Welfare, after review and approval by the Novartis Institutional Animal Care and Use Committee (East Hanover, NJ, USA). Cardiac tissues from one male adult human donor were purchased from the International Institute for the Advancement of Science (USA).

### Cell Culture, Transfection and Reporter Assay

Human pulmonary artery smooth muscle cells (HPASM cells, Lonza CC-2581 lot# 7F33558) were cultured according to supplier instructions. The day of transfection, cells were trypsinized, rinsed with HEPES, and resuspended in 500 µl Nucleofector solution (Lonza, CH) supplemented with 50 nM of microRNA-mimics (Dharmacon, Lafayette, CO) according to Amaxa transfection protocol A-033. After 24 h, cells were lysed in Qiazol to retrieve RNA.

Regions of interest of mRNAs 3′UTR (Figure S7 and Materials and Methods S1) were cloned by synthesis in pmirGLO Dual-Luciferase microRNA Target Expression Vector (Promega, Madison, WI). Target seeds were mutated using QuikChange II Site-Directed Mutagenesis Kits (Agilent Technologies, Santa Clara, CA). Primers were designed according to manufacturer instructions. Primer sequences are available in Materials and Methods S1.

Hek293 cells were cultured according to supplier instructions. Cells (2×10^5^ per well, in 24-wells plates) were co-transfected with 20 ng of pmiR-GLO reporter construct of interest and synthetic microRNA mimics (40 nM) (Dharmacon, Lafayette, CO) using Lipofectamine 2000 (Invitrogen, Carlsbad, CA). A *C. elegans* mimic with no affinity for mammalian targets was used as negative control. Dual-GLO Luciferase Assay System (Promega, Madison, WI) was used to obtain the luciferase/Renilla relative luminescence, according to manufacturer instructions. Data were averaged for n = 4 in 3 independent experiments.

### RNA Isolation

Cardiac tissue, sectioned and snap frozen as described in the previous section, was homogenized in Qiazol (Qiagen) reagent according to the manufacturer’s instructions. Organic extraction of RNA was achieved by adding chloroform and high speed centrifugation at 4°C. RNA-containing upper aqueous phase was mixed with 1 volume of 70% EtOH and loaded into RNeasy minispin columns and briefly spinned at room temperature according to miRNeasy mini kit instructions (Qiagen). The content of this filter-column was then processed to obtain total RNA, while the first flow through, enriched in <200 nt RNA (small RNA fraction) was then processed through an RNeasy Clean-up column, according to manufacturer instructions (Qiagen, MD). Small RNA was eluted in 15 µl of nuclease free water, quantified via 260 nm absorbance and quality checked using the Bioanalyzer small RNA chips.

### Experimental Strategy for Heart Structure-specific Transcriptomic Profiling

Different cardiac structures were dissected by hand from at least 3 Wistar rats, Beagle dogs and cynomolgus monkeys, as indicated in Figure 1. Both small RNA (<200 nt) and mRNA were isolated from each sample. Messenger RNA transcriptomes were generated via Affymetrix Gene Chip technology for all samples (Figure 1). Sequencing reads were mapped to miRBase version 19 containing 2042 human, 1281 murine and 289 canine entries. In parallel, rat microRNAs were also profiled using a TLDA qPCR approach. On average, a Spearman correlation of 0.835±0.016 was observed when comparing the TLDA with the microRNA sequencing data set, confirming the reliability of the deep sequencing approach (Figure S4).

### Gene Expression Profiling

Processing of total RNA and Gene Chip experiments were conducted as recommended by the manufacturer (Affymetrix, Santa Clara, CA). Messenger RNAs isolated from rat, canine and cynomolgus were hybridized on 3′IVT arrays Rat230_2, Canine_2 and HG-U133_Plus_2 respectively. Corresponding.cel are available in Tables S11, S12 and S13 respectively.

HPASM cells endogenous genes expression was obtained using Taqman assays for the corresponding human transcript, according to manufacturer instructions. Briefly 300 ng of RNA were reverse transcribed to cDNA using the High Capacity Reverse Transcription kit. Two µl of cDNA were used in a 20 µl qPCR reaction in 7900 HT machines (all Applied Biosystems, Life Technologies, Carlsbad, CA), 18S was used as reference housekeeping gene.

### MicroRNA Next Generation Sequencing

Indexed microRNA sequencing libraries were prepared from 100 ng small RNA using TruSeq Small RNA Sample Preparation Kits (Illumina). After PCR amplification multiplexed libraries were generated by equimolary pooling of individual libraries. The multiplexed libraries were size selected on 6% TBE-PAGE gels (Invitrogen). The libraries were loaded on HiSeq Single Read Flow Cells v1.5 using TruSeq Single-Read Cluster Generation Kits (v2). The sequencing runs were done on HiSeq 2000 instruments.

### TLDA qPCR

Three hundred and eighty rodent microRNAs were profiled using the Taqman Low Densitiy Array (TLDA) technology (Applied Biosystems, Life Technologies, Carlsbad, CA) according to the manufacturer instructions (Applied Biosystems, Life Technologies, Carlsbad, CA). Mammalian snRNA U6 was used as housekeeping gene for data normalization.

### Localization of microRNAs by ISH

Detection of microRNA by ISH was performed using double-digoxigenin labeled miRCURY LNA™ probe provided by Exiqon A/S (Vedbaek, Denmark).

ISH on rat heart tissue section was done using the fully automated instrument Ventana Discovery Ultra® (Roche Diagnostics, Rotkreuz, Switzerland). All chemicals were also provided by Roche Diagnostics except the “microRNA ISH optimization kit” provided by Exiqon A/S. Briefly, formalin fixed paraffin embedded sections were de-paraffinized and rehydrated under solvent-free conditions (EZprep solution) and pretreated by enzymatic digestion (Proteinase K at 12 µg/ml for 16 minutes at 37°C). Hybridization was performed adding to each slide 50 nM of DIG-LNA probe diluted in the Exiqon hybridization buffer and incubated for 3 hrs. After hybridization, sections were washed on stringency conditions (see Table S1 for details).

DIG-label LNA probe detection was performed using an Alkaline Phosphatase-conjugated Sheep anti-digoxigenin antibody (Roche Diagnostics) diluted 1/500 in antibody diluent. Antibody incubation was carried out for 30 min at 37°C followed by chromogenic detection using BlueMap™ Kit (Ventana, Roche) with a substrate incubation time of 6 hrs.

Counterstaining using ISH nuclear fast red was performed for 2 min. Sections were mounted in Glycerol-gelatin mounting medium (Sigma-Aldrich Chemie GmbH, Buchs, Switzerland) and post-mounted using Pertex™ (Histolab).

### Data Analysis of Gene Expression Profiles and microRNAs Sequencing

The generated “.cel” files were loaded in Genespring 11.5.1 (Agilent Technologies, Santa Clara, CA) and further processed according to the standard RMA workflow. Expression data were analyzed at the gene level. Preferentially enriched gene lists were generated by retaining the top 10^th^ percentile most expressed genes in all replicates of at least one structure. Genes that were 5-fold more expressed in one cardiac structure *vs.* the median of the other tissue groups were retained.

Illumina NGS reads were aligned to the respective miRBase v.19 [64] reference sequences for the different species and to the *in silico* computed sequences of their precursor molecules using the Bowtie short-read aligner [65]. The microRNA abundance was quantified using an in-house NGS analysis pipeline, counting aligned reads for each microRNA down-weighted by the number of equivalent alignments of the same read to any of the other microRNA species. Those weighted counts where subsequently normalized by a robust estimate of the individual sequencing library sizes based on the median of the ratios of observed counts for a sample *j* against a pseudo-reference sample obtained by taking the geometric mean of microRNA *i* across all samples: 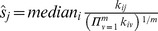 [66]. Normalized expression microRNA values were stored in Tables S8 for rat, S9 for dog and S10 for cynomolgus monkey. MicroRNAs hierarchical Euclidean centroid-linkage clustering and distance matrix were plotted using MeV package [67]. The normalized read counts were averaged per structure and the top 10th percentile most expressed microRNAs in at least one structure were compared across the other tissue groups of the same species in order to determine those preferentially expressed in each structure (average expression fold change >2, top 10th percentile expressed). All mRNA expression values were computed using the standard Bioconductor RMA normalization and quantification algorithm [68]. The mouse microRNA target predictions were extracted from TargetScan (Version 5.2) [69] and translated to the rat and dog genomes by homology mapping based on the ENSEMBL gene orthology predictions. Spearman rank correlation factors ranging from +1 to -1 were computed based on the expression values of all those predicted microRNA/mRNA pairs across the different cardiac tissues. Thus negative values are obtained for those target pairs where higher microRNA expression in certain heart tissues is contrasted with lower mRNA expression in those same tissues, or vice versa. Such microRNA/mRNA target pairs will be referred to as anti-correlated (Tables S5, S6 and S7 for rat, dog and cynomolgus monkey respectively).

The degree of correlation between qPCR and microRNA sequencing profiles was obtained by computing Pearson and Spearman correlation factors (r and r_s_ respectively) for each sample pair and for the microRNAs detected by both technologies.

## Supporting Information

Figure S1 Cardiac structures are similarly clustered by microRNA and mRNA profiles of dog (A) and cynomolgus (B), according to their histological characteristics. microRNAs were mapped against miRbase17. Red circles: myocardial tissue (apex, left and right ventricle, septum, papillary muscle). Blue circles: left and right atrium. Grey circles: cardiac valves.
(TIF)

Figure S2 Correlation of 4 cardiac disease relevant genes with putative targeting microRNA in canine. Timp3 and miR-1 (A), Rbm24 and miR-125b-5p (B), Tgfbr2 and miR-204 (C), Csnk2a2 and miR-208b (D). Green curve represents log2 normalized intensity for the indicated probe set. Black curve represents log2 scaled and normalized microRNA read counts. Three replicates are plotted for each structure.
(TIF)

Figure S3 Correlation of 4 cardiac disease relevant genes with putative targeting microRNA in cynomolgus monkey. Timp3 and miR-1 (A), Rbm24 and miR-125b-5p (B), Tgfbr2 and miR-204 (C), Csnk2a2 and miR-208b (D). Green curve represents log2 normalized intensity for the indicated probe set. Black curve represents log2 scaled and normalized microRNA read counts. Three replicates are plotted for each structure.
(TIF)

Figure S4 Robustness of microRNA sequencing and comparison to qPCR. Data obtained with microRNA sequencing or TLDA cards are compared for each rat cardiac sample. rs = Spearman’s rank correlation, r = Pearson’s correlation. On average, 247 microRNAs were detected on the TLDA.
(TIF)

Figure S5 Conservation of (A) Timp3 miR-1/206 targeting seed, (B) Rbm24 miR-125b-5p targeting seed, (C) Tgfbr2 miR-204 targeting seed, (D) Csnk2a2 miR-208b targeting seed. Rno: Rattus norvegicus. Hsa: Homo sapiens. Ptr: Pan trogloditus. Mml: Macaca mulatta. Mmu: Mus musculus. Cfa: Canis familiaris.
(TIF)

Figure S6 Distribution of miR-1, miR-125b-5p, miR-204 and miR-208b in the cardiac structures in 1 human donor. Axes represent fold change vs. apex. A, apex; LA, left atrium; LV, left ventricle; PM, papillary muscle; RA, right atrium; RV, right ventricle; S, septum; V, valve.
(TIF)

Figure S7 Cloning of 3′ UTRs of Timp3, Csnk2a2, Rbm24, Rbm38, Tgfbr2 and Akap2 in pmiR-GLO. Yellow arrows indicate ORFs. Grey arrows identify the region surrounding the microRNA Targetscan targeting site cloned in pmiR-GLO.
(TIF)

Table S1 LNA probe hybridization conditions.
(XLS)

Table S2 Rat cardiac structure enriched microRNAs.
(XLS)

Table S3 Canine cardiac structure enriched microRNAs.
(XLS)

Table S4 cynomolgus monkey cardiac structure enriched microRNAs.
(XLS)

Table S5 miRNA/mRNA correlations in Rat.
(ZIP)

Table S6 miRNA/mRNA correlations in Canine.
(ZIP)

Table S7 miRNA/mRNA correlations in Cynomolgus monkey.
(ZIP)

Table S8 Normalized miR-Seq data Rat.
(ZIP)

Table S9 Normalized miR-Seq data Canine.
(ZIP)

Table S10 Normalized miR-Seq data cynomolgus monkey.
(ZIP)

Table S11 mRNA expression data_Rat.
(ZIP)

Table S12 mRNA expression data_Canine.
(ZIP)

Table S13 mRNA expression data_Cynomolgus monkey.
(ZIP)

Materials and Methods S1 Cloned regions of Timp3, Rbm24, Rbm38, Akap2, Tgfbr2 and Csnk2a2.
(DOC)
