# Supplementary figures and images for: Heart Structure-Specific Transcriptomic Atlas Reveals Conserved microRNA-mRNA Interactions
Source: PLoS One. 2013 Jan 2;8(1):e52442. doi: 10.1371/journal.pone.0052442 (PMC3534709; doi:10.1371/journal.pone.0052442)

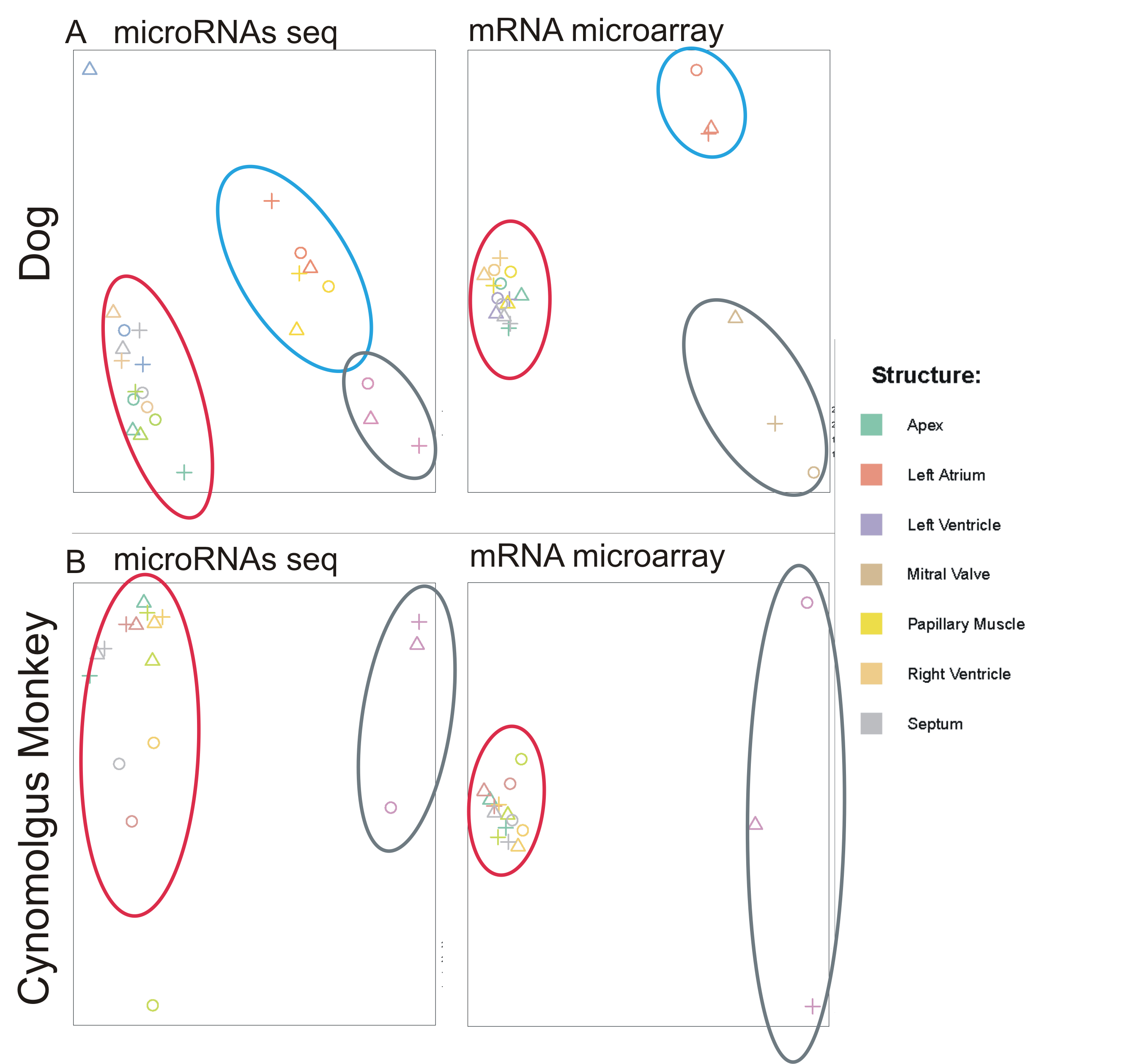

Supplement: Figure S1 — Cardiac structures are similarly clustered by microRNA and mRNA profiles of dog (A) and cynomolgus (B), according to their histological characteristics. microRNAs were mapped against miRbase17. Red circles: myocardial tissue (apex, left and right ventricle, septum, papillary muscle). Blue circles: left and right atrium. Grey circles: cardiac valves. (TIF) [file pone.0052442.s001.tif]

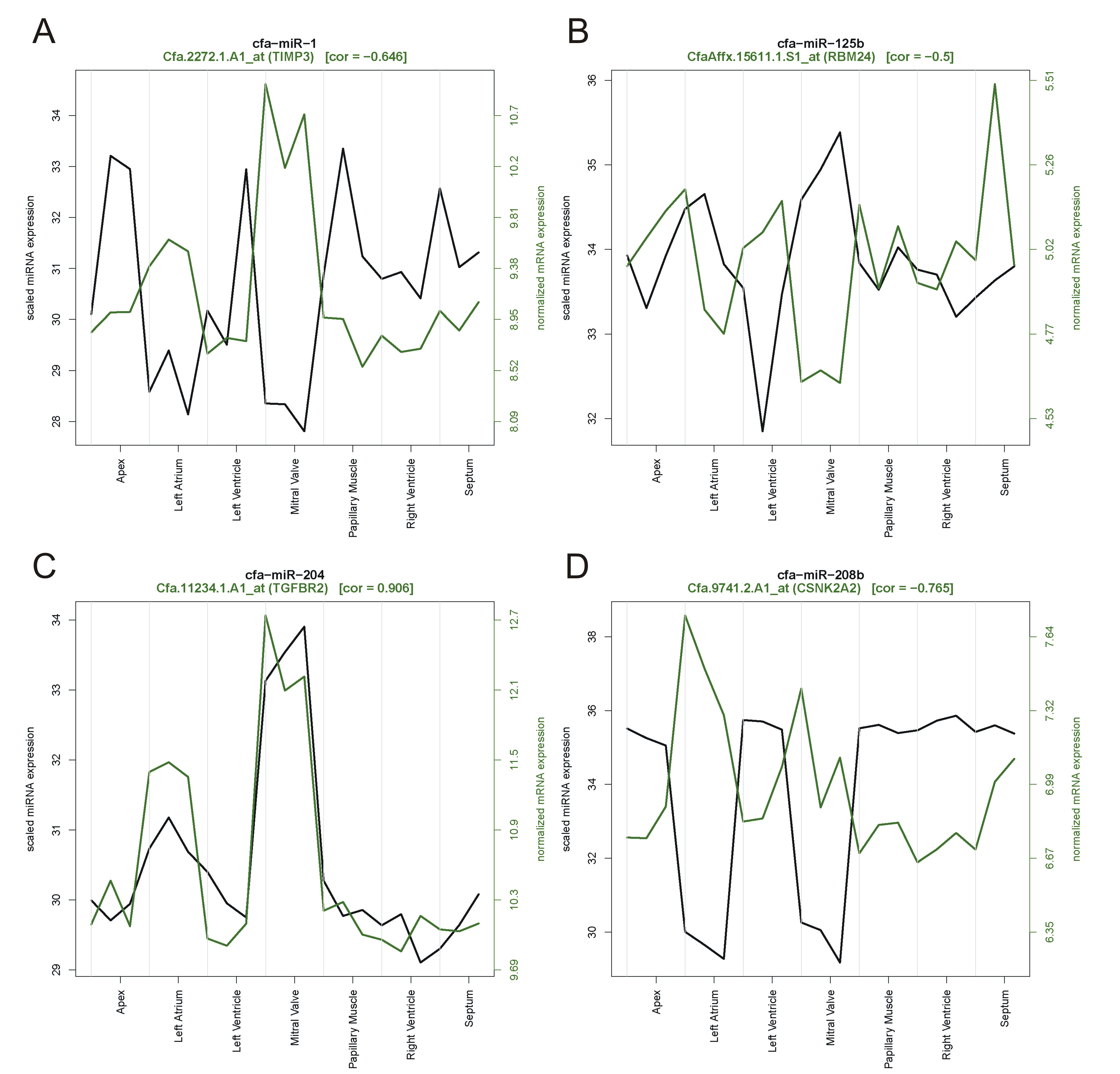

Supplement: Figure S2 — Correlation of 4 cardiac disease relevant genes with putative targeting microRNA in canine. Timp3 and miR-1 (A), Rbm24 and miR-125b-5p (B), Tgfbr2 and miR-204 (C), Csnk2a2 and miR-208b (D). Green curve represents log2 normalized intensity for the indicated probe set. Black curve represents log2 scaled and normalized microRNA read counts. Three replicates are plotted for each structure. (TIF) [file pone.0052442.s002.tif]

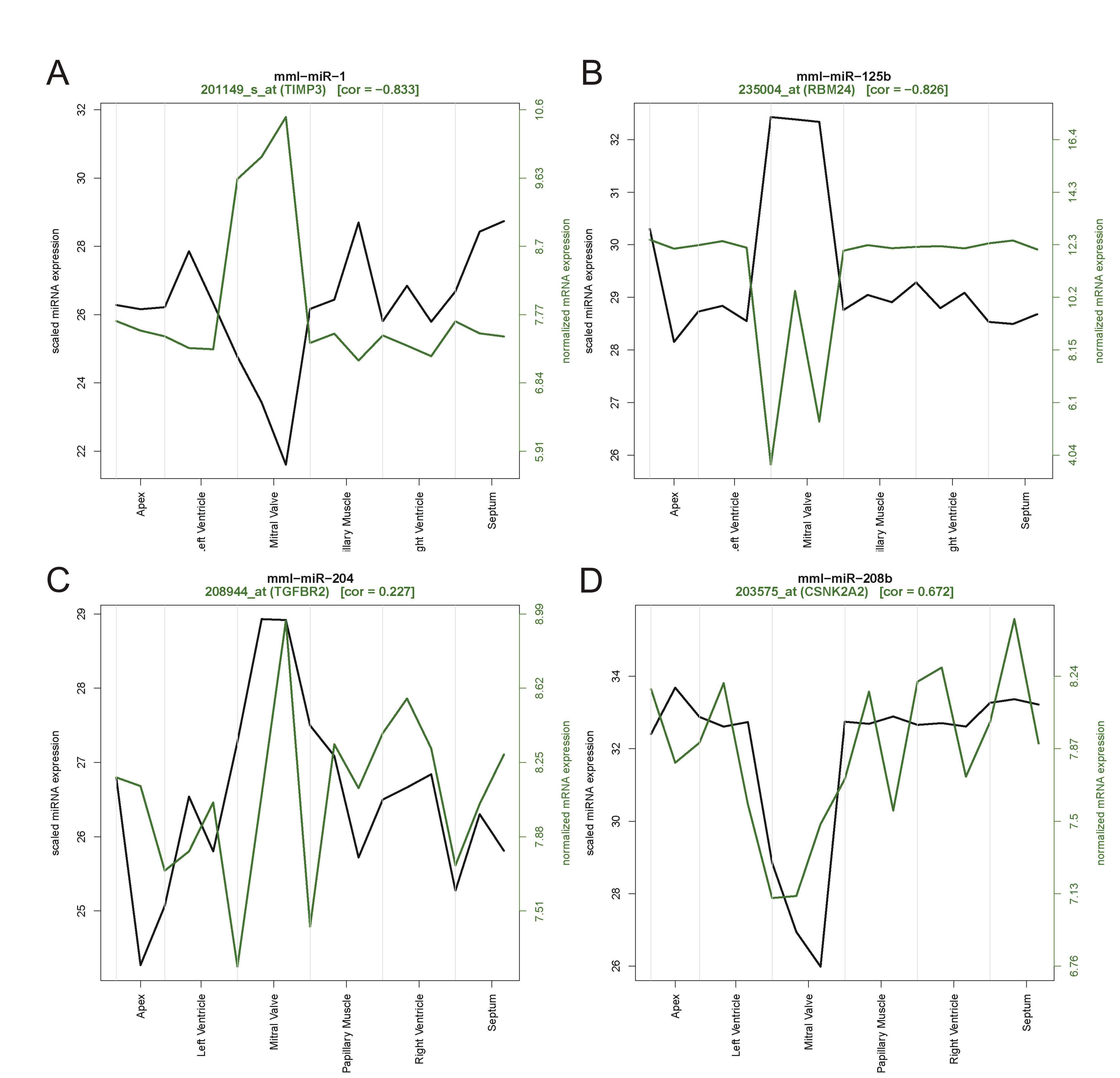

Supplement: Figure S3 — Correlation of 4 cardiac disease relevant genes with putative targeting microRNA in cynomolgus monkey. Timp3 and miR-1 (A), Rbm24 and miR-125b-5p (B), Tgfbr2 and miR-204 (C), Csnk2a2 and miR-208b (D). Green curve represents log2 normalized intensity for the indicated probe set. Black curve represents log2 scaled and normalized microRNA read counts. Three replicates are plotted for each structure. (TIF) [file pone.0052442.s003.tif]

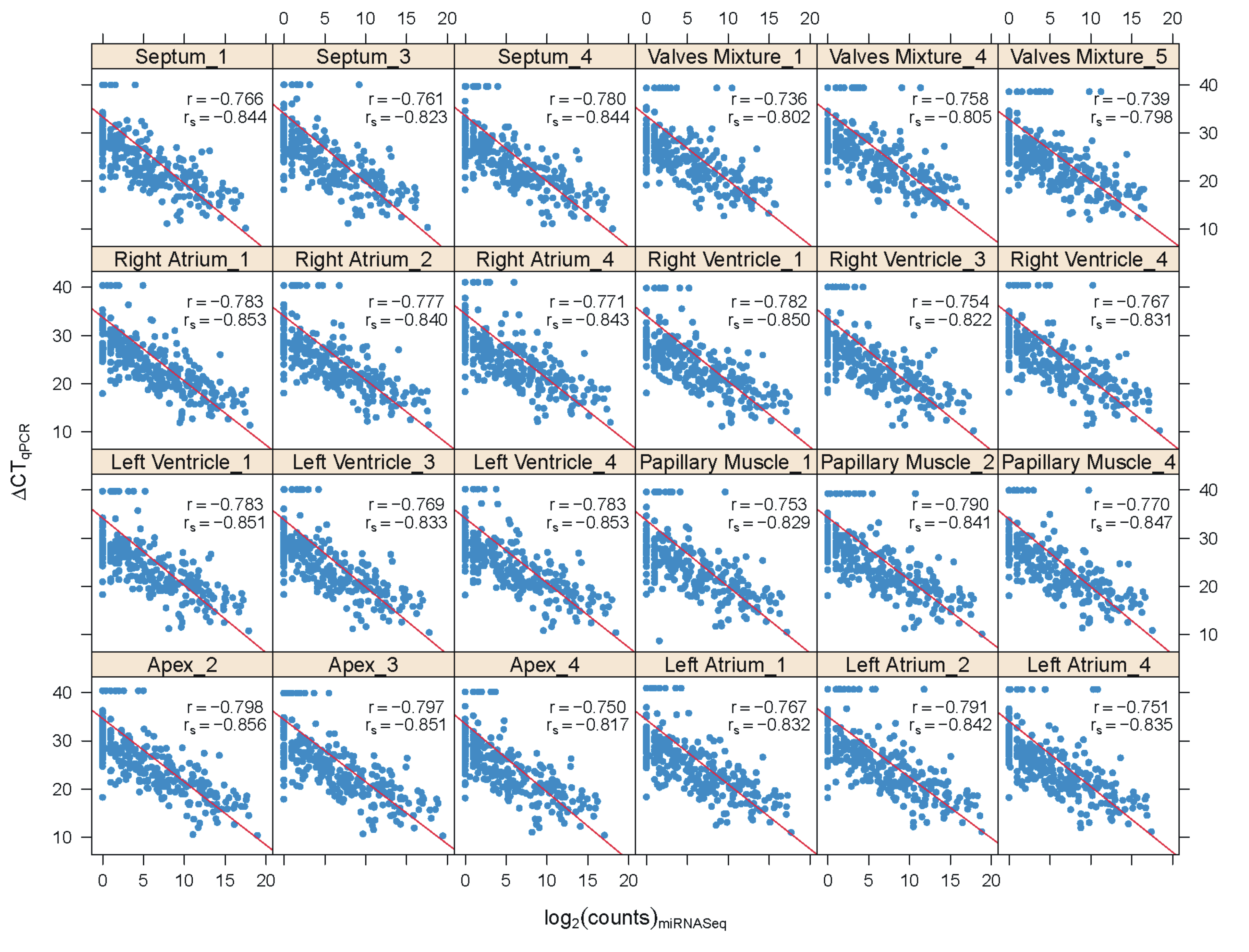

Supplement: Figure S4 — Robustness of microRNA sequencing and comparison to qPCR. Data obtained with microRNA sequencing or TLDA cards are compared for each rat cardiac sample. rs = Spearman’s rank correlation, r = Pearson’s correlation. On average, 247 microRNAs were detected on the TLDA. (TIF) [file pone.0052442.s004.tif]

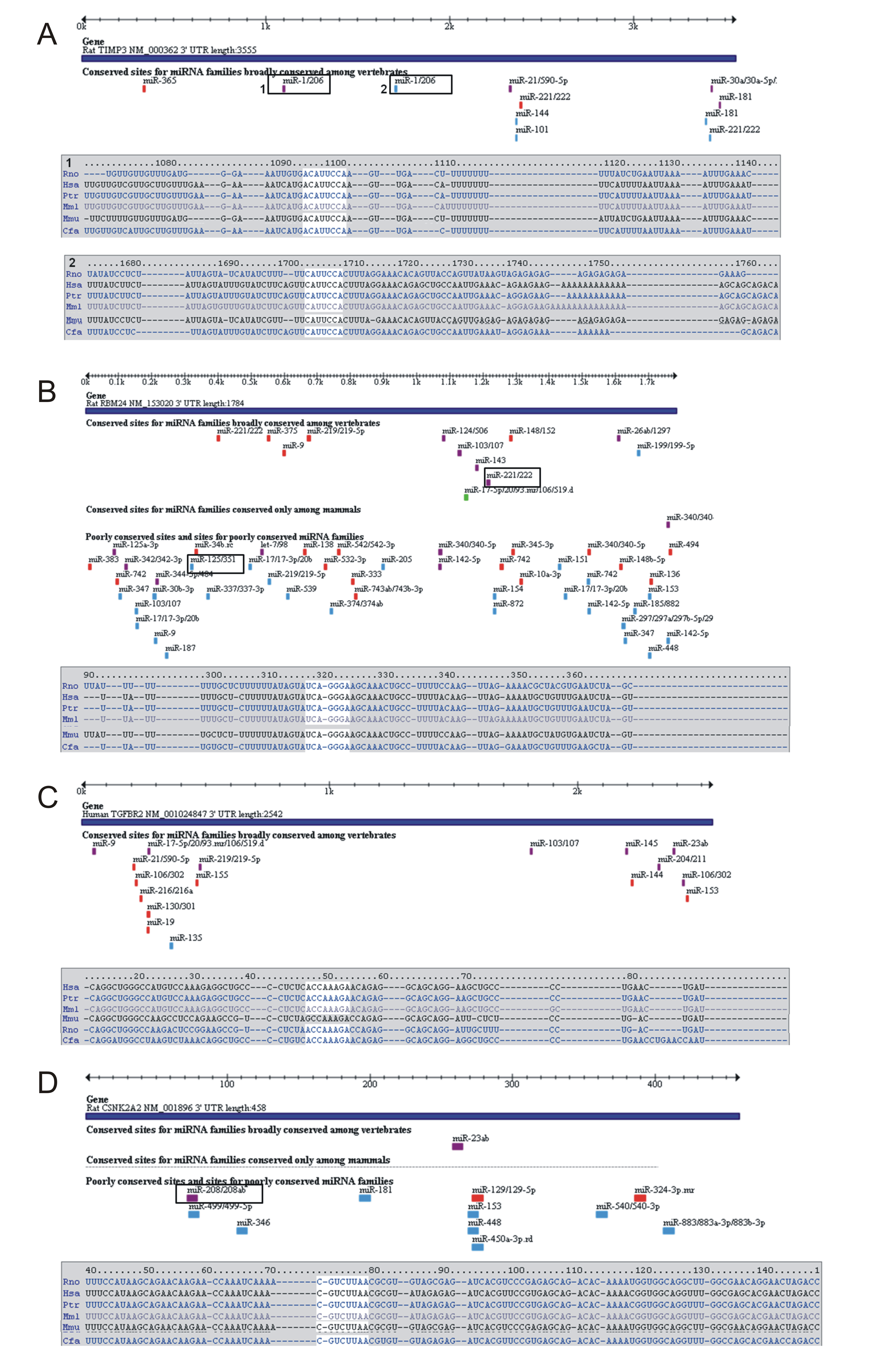

Supplement: Figure S5 — Conservation of (A) Timp3 miR-1/206 targeting seed, (B) Rbm24 miR-125b-5p targeting seed, (C) Tgfbr2 miR-204 targeting seed, (D) Csnk2a2 miR-208b targeting seed. Rno: Rattus norvegicus. Hsa: Homo sapiens. Ptr: Pan trogloditus. Mml: Macaca mulatta. Mmu: Mus musculus. Cfa: Canis familiaris. (TIF) [file pone.0052442.s005.tif]

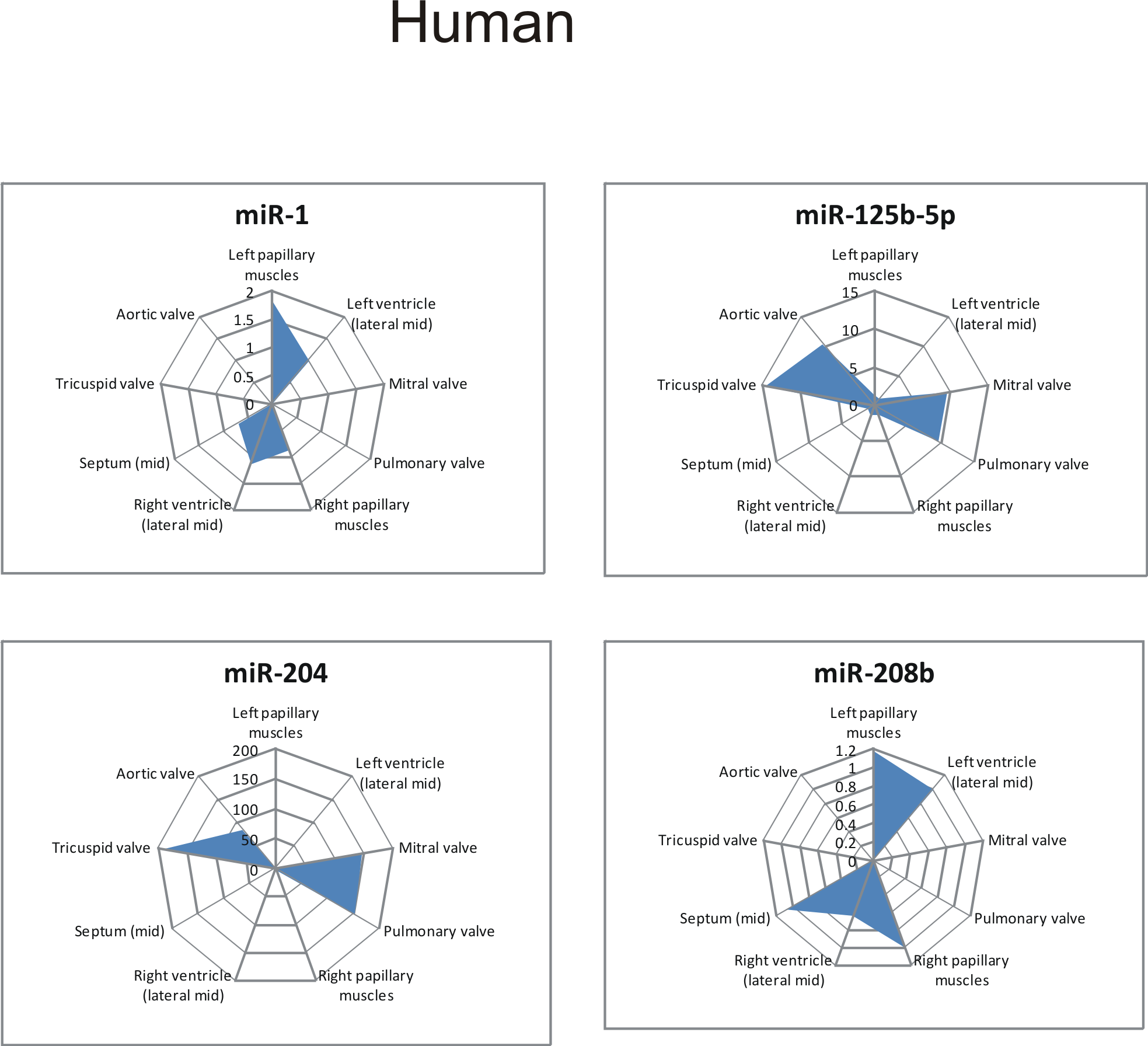

Supplement: Figure S6 — Distribution of miR-1, miR-125b-5p, miR-204 and miR-208b in the cardiac structures in 1 human donor. Axes represent fold change vs. apex. A, apex; LA, left atrium; LV, left ventricle; PM, papillary muscle; RA, right atrium; RV, right ventricle; S, septum; V, valve. (TIF) [file pone.0052442.s006.tif]

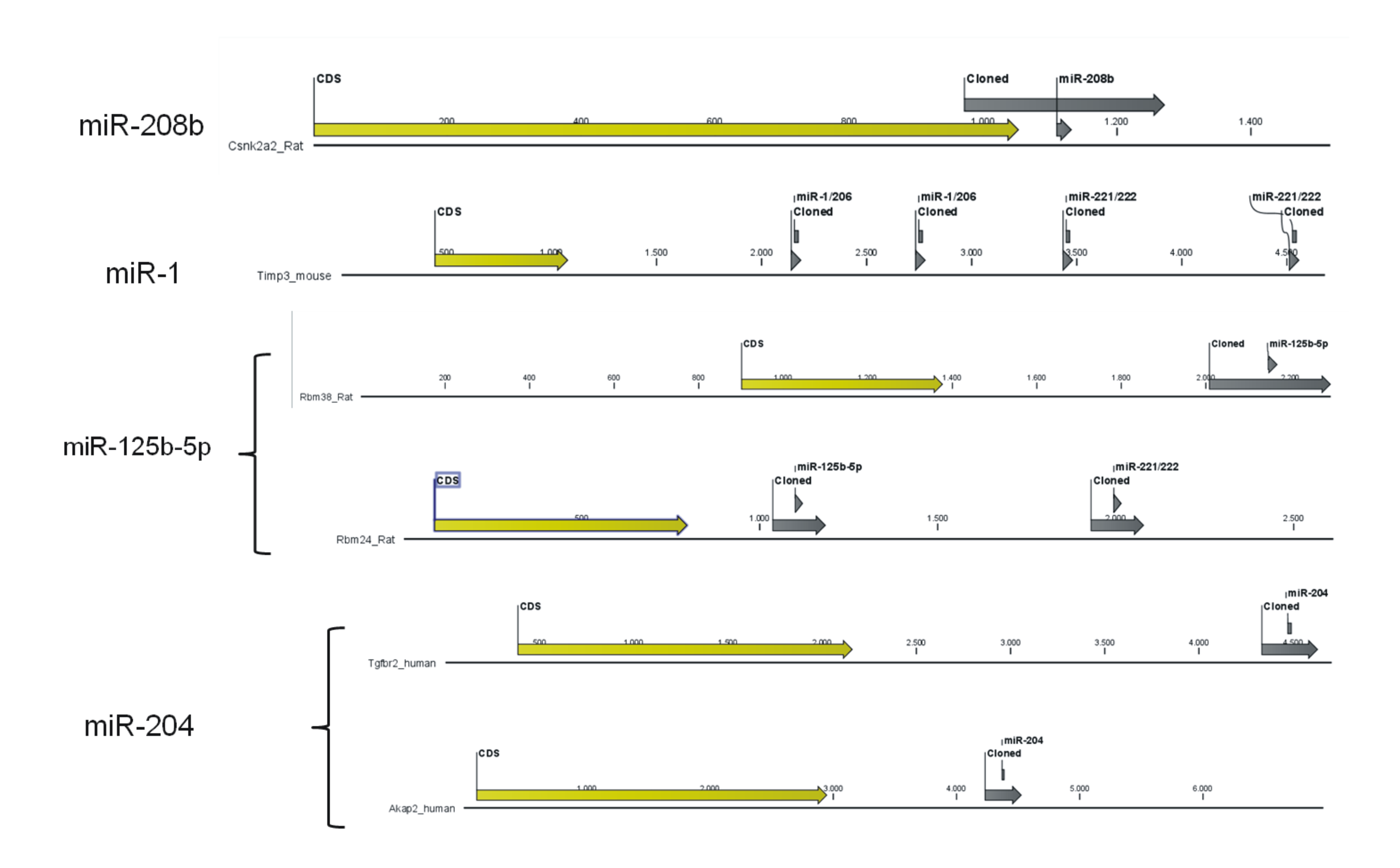

Supplement: Figure S7 — Cloning of 3′ UTRs of Timp3, Csnk2a2, Rbm24, Rbm38, Tgfbr2 and Akap2 in pmiR-GLO. Yellow arrows indicate ORFs. Grey arrows identify the region surrounding the microRNA Targetscan targeting site cloned in pmiR-GLO. (TIF) [file pone.0052442.s007.tif]
