# Supplementary material for: Heart Structure-Specific Transcriptomic Atlas Reveals Conserved microRNA-mRNA Interactions
Source: PLoS One. 2013 Jan 2;8(1):e52442. doi: 10.1371/journal.pone.0052442 (PMC3534709; doi:10.1371/journal.pone.0052442)
Supplement: Materials and Methods S1 — Cloned regions of Timp3, Rbm24, Rbm38, Akap2, Tgfbr2 and Csnk2a2. (DOC) [file pone.0052442.s021.doc]

# Material and Methods S1

## Cloned regions of Timp3, Rbm24, Rbm38, Akap2, Tgfbr2 and Csnk2a2

Relative position of cloned stretches is shown in Figure S7. Mutations are in indicated in bold.

Timp3 WT 5’-3’ (Murine NM_011595.2):

Agtttaatctttttttcttttgttgtttgatgggaaattgtgacattccaagttgacttttttttattatctgaacagattatgggtatttatcctctattagtatcatatcgttttcattccactttagaaacacagttaccagttgagtatttcacctgttcacttactgtataatttaaaatcatttatgtagctgagacactttgatacttcaattatatcgagaaaaccccctctagaaggaatgtatttgttgctaaattttgtagcactgtttacagttttcctccatgtta

Timp3 S1-S2 MUT 5’-3’ (Murine NM_011595.2):

AGTTTAATCTTTTTTTCTTTTGTTGTTTGATGGGAAATTGTG**tgtaagg**AAGTTGACTTTTTTTTATTATCTGAACAGATTATGGGTATTTATCCTCTATTAGTATCATATCGTTTT**gtaaggt**CTTTAGAAACACAGTTACCAGTTGAGTATTTCACCTGTTCACTTACTGTATAATTTAAAATCATTTATGTAGCTGAGACACTTTGATACTTCAATTATATCGAGAAAACCCCCTGAAGAAGGAATGTATTTGTTGCTAAATTTTGTAGCACTGTTTACAGTTTTCCTCCATGTTA

Rbm24 WT 5’-3’ (Rat NM_001191100):

Aggacacgatggcaggggtacccatgggacttcacttttgtatggggatttttattttttttgctcttttttatagtatcagggaagcaaactgccttttccaagttagaaaacgctacgtgaatctagctgaaccagggaatacggagtctctaaaggaaggaactttagaagtgacactgtaaaattatgtattcatctcatggcataagttattcagtaggtctagatgtagcatattaaatattaacctattcaactaaagatgttggctttggatttatttaaattcttatgtgc

Rbm24 MUT 5’-3’ (Rat NM_001191100):

GAGAGGACACGATGGCAGGGGTACCCATGGGACTTCACTTTTGTATGGGGATTTTTATTTTTTTTGCTCTTTTTTATAGTA**agtccct**AGCAAACTGCCTTTTCCAAGTTAGAAAACGCTACGTGAATCTAGCTGAACCAGGGAATACGGAGTCTCTAAAGGAAGGAACTTTAGAAGTGACACTGTAAAATTATGTATTCATCTCATGGCATAAGTTATTCAGTAGGTCTAGatgtagcATATTAAATATTAACCTATTCAACTAAAGATGTTGGCTTTGGATTTATTTAAATTCTTATGTGC

Rbm38 WT 5’-3’ (Rat NM_001108965.1):

CACCCAGCCCCCCATTGTCACCTGTACCAGTCTGGAGCATGACCTAGCTGCACAGGGAGAAGGTCCAGCCCACACACGTCTGGGAGGGGAGGAGTGGGCATCTCTGGCAGCATGGGCTGCCCACAGGCTGGGCAGTGGGTCCCTCCACACCTACCTCAGGGAGCCAGTGTGTAGGCACTCCACAGCATGCCTGTTAGTCAAGGCCTGGAGGACAGAGGGAGTAATGTTATAATAATATTTTTATTAGAATGTTCTGATTATAAAAAATAAAACTTGTTTTCTTTAAAG

Akap2 WT 5’-3’ (Human NM_001004065):

ATTGTGTGTTTGTAAAATGTGTATGTTCATGAGTAAGGGTGTGTGTGTGTGTGTATTAAAATTCCAGAGTGACCGTGGCACTTGGGTGTACAGGTAATTCCTCCAGAGCTGTTTGCTGGCTTCAGGAGTGGAGTGAGAATTTCTTTTTTATGAAAAGGGATATAAAGGCACCGAGCTGATGCAGTATTTGTAATATTAAGTTGACCTAACAAGGTATTTGCATGAGTCACAATTACAAAGTTTTGAGCGGTTTTGTAATTTGACATTTAGGAGAGTCTCCTATTTATTCTCATACTTTAC

Akap2 MUT 5’-3’ (Human NM_001004065):

ATTGTGTGTTTGTAAAATGTGTATGTTCATGAGTAAGGGTGTGTGTGTGTGTGTATTAAAATTCCAGAGTGACCGTGGCACTTGGGTGTACAGGTAATTCCTCCAGAGCTGTTTGCTGGCTTCAGGAGTGGAGTGAGAATTTCTTTTTTATGA**tttccct**TATAAAGGCACCGAGCTGATGCAGTATTTGTAATATTAAGTTGACCTAACAAGGTATTTGCATGAGTCACAATTACAAAGTTTTGAGCGGTTTTGTAATTTGACATTTAGGAGAGTCTCCTATTTATTCTCATACTTTAC

Tgfbr2 WT 5’-3’ (Human NM_001024847):

CTCACTTCTGGGTTATCAGCATAAACTGGAATGTAGTGTCAGAGGATACTGTGGCTTGTTTTGTTTATGTTTTTTTTTCTTATTCAAGAAAAAAGACCAAGGAATAACATTCTGTAGTTCCTAAAAATACTGACTTTTTTCACTACTATACATAAAGGGAAAGTTTTATTCTTTTATGGAACACTTCAGCTGTACTCATGTATTAAAATAGGAATGTGAATGCTATATACTCTTTTTATATCAAAAGTCTCAAGCACTTATTTTTATTCTATGCATTGTTTGTCTTTTACATAAATAAAA

Tgfbr2 MUT 5’-3’ (Human NM_001024847):

CTCACTTCTGGGTTATCAGCATAAACTGGAATGTAGTGTCAGAGGATACTGTGGCTTGTTTTGTTTATGTTTTTTTTTCTTATTCAAGAAAAAAGACCAAGGAATAACATTCTGTAGTTCCTAAAAATACTGACTTTTTTCACTACTATACAT**tttccct**AAGTTTTATTCTTTTATGGAACACTTCAGCTGTACTCATGTATTAAAATAGGAATGTGAATGCTATATACTCTTTTTATATCAAAAGTCTCAAGCACTTATTTTTATTCTATGCATTGTTTGTCTTTTACATAAATAAAA

Csnk2a2 WT 5’-3’ (Rat NM_001107409):

CTTCTACCCGGTGGTGAAGGAGCAGTCCCAGCCTTGTGCTGAGAACACCGTGCTTTCCAGTGGTCTCACCGCAGCACGATGAAGCCTGGGGAATCGACGGTCTGTTGCGGTTCCTCCCACTTTTCCATAAGCAGAACAAGAACCAAATCAAAACGTCTTAACGCGTGTAGCGAGATCACGTCCCGAGAGCAGACACAAAATGGTGGCAGGCTTGGCGAACAGGAACTAGACCACCCGAAGGGCAGCCCACCACCGTAAATCAGACCTCACTTCCGAATGTAAAAGGTTCACATGCCTTTG

Csnk2a2 MUT 5’-3’ (Rat NM_001107409):

CTTCTACCCGGTGGTGAAGGAGCAGTCCCAGCCTTGTGCTGAGAACACCGTGCTTTCCAGTGGTCTCACCGCAGCACGATGAAGCCTGGGGAATCGACGGTCTGTTGCGGTTCCTCCCACTTTTCCATAAGCAGAACAAGAACCAAATCAAAA**gcagaat**ACGCGTGTAGCGAGATCACGTCCCGAGAGCAGACACAAAATGGTGGCAGGCTTGGCGAACAGGAACTAGACCACCCGAAGGGCAGCCCACCACCGTAAATCAGACCTCACTTCCGAATGTAAAAGGTTCACATGCCTTTG
